# Shikonin, dually functions as a proteasome inhibitor and a necroptosis inducer in multiple myeloma cells

**DOI:** 10.3892/ijo.2014.2804

**Published:** 2014-12-19

**Authors:** NAOKO WADA, YAWARA KAWANO, SHIHO FUJIWARA, YOSHITAKA KIKUKAWA, YUTAKA OKUNO, MASAYOSHI TASAKI, MITSUHARU UEDA, YUKIO ANDO, KAZUYA YOSHINAGA, MASAKI RI, SHINSUKE IIDA, TAKAYUKI NAKASHIMA, YUKIMASA SHIOTSU, HIROAKI MITSUYA, HIROYUKI HATA

**Affiliations:** 1 Department of Hematology, Kumamoto University Hospital, Kumamoto, Japan; 2 Department of Medical Oncology, Dana-Farber Cancer Institute, Boston, MA, USA; 3 Department of Hematology and Oncology, Nagoya City University Graduate School of Medical Sciences, Nagoya, Japan; 4 Department of Neurology, Graduate School of Medical Sciences, Kumamoto University, Kumamoto, Japan; 5 Division of Informative Clinical Sciences, Graduate School of Health Sciences, Kumamoto University, Kumamoto, Japan; 6 Department of Anatomy, Graduate School of Health Sciences, Kumamoto University, Kumamoto, Japan; 7 Research Functions Unit, Kyowa Hakko Kirin Co., Ltd., Japan; 8 Translational Oncology, Kyowa Hakko Kirin California Inc., La Jolla, CA, USA

**Keywords:** multiple myeloma, apoptosis, necroptosis, heat shock protein, proteasome

## Abstract

Shikonin (SHK), a natural small agent (MW 288.3), reportedly induces cell death in various tumor cells. We have found that SHK also exerts potent cytocidal effects on human multiple myeloma (MM) cells, but its anticancer mechanism in MM cells remains to be elucidated. SHK at 2.5–5 μM induced apoptosis in seven MM cell lines, including the bortezomib-resistant cell line KMS11/BTZ. The IC_50_ value of SHK against KMS11/BTZ was comparable to that of a parental cell line KMS11 (1.1 and 1.56 μM, respectively). SHK induces accumulation of ubiquitinated proteins and activates XBP-1 in MM cells, suggesting that SHK functions as a proteasome inhibitor, eventually inducing ER stress-associated apoptosis. SHK increases levels of HSP70/72, which protects cells from apoptosis, and exerts greater cytocidal effects in combination with the HSP70/72 inhibitor VER-155008. At higher concentrations (10–20 μM), SHK induced cell death, which was completely inhibited by a necroptosis inhibitor, necrostatin-1 (Nec-1), while the cytocidal activity was unaffected by Z-VAD-FMK, strongly suggesting that cell death is induced by SHK at high concentrations through necroptosis. The present data show for the first time that SHK induces cell death in MM cells. SHK efficiently induces apoptosis and combination of heat shock protein inhibitor with low dose SHK enhances apoptosis, while high dose SHK induces necroptosis in MM cells. These findings together support the use of SHK as a potential therapeutic agent for MM.

## Introduction

Despite recent advances in developing therapeutic strategy for multiple myeloma (MM), MM still remains incurable and thus a novel therapeutic approach is urgently needed (1). By screening natural compound libraries, we found that shikonin (SHK), a natural naphthoquinone derivative isolated from the root of *Lithospermum erythrorhizon*, efficiently induced cell death in MM cells.

SHK has been reported to induce cell death in various tumor cell lines, such as breast cancer, leukemia and prostate cancer (2–6). Recent report showing efficacy of SHK to human promyelocytic leukemia cells by interacting with thioredoxin reductase may promise efficacy of SHK to human hematopoietic tumors, not only myeloma cells, through multiple activities.

However, there are no reports showing the efficacy of SHK in MM cells. Although mechanisms by which SHK regulates cell death have not been fully analyzed, SHK has been shown to induce activation of caspases (4,5) by modulating Bcl-2 family proteins, p27 and p53 (7), which eventually leads to apoptosis. Involvement of reactive oxygen species (ROS) (8,9) and the NFκB signaling pathway (10) in SHK-mediated apoptosis have also been reported. SHK also functions as a proteasome inhibitor (11) and topoisomerase I inhibitor (12). SHK has also been demonstrated to induce the molecular chaperone heat shock protein 70 (HSP70) (13,14).

Previous studies have implicated a function for HSP70 in MM cells. Inhibition of HSP70 reversed drug resistance and induced apoptosis in MM cells (15–17), indicating a contribution of HSP70 to the survival of MM cells. However, the role of HSP70 in MM cells has not been extensively analyzed.

Interestingly, SHK is also an inducer of necroptosis in osteosarcoma and glioma cells (18,19). Necroptosis (programmed necrosis) is a type of cell death distinct from apoptosis (20). Necroptosis depends on the kinase activities of receptor-interacting proteins 1 and 3 (RIP1 and RIP3) and is specifically inhibited by the small molecule necrostatin-1 (Nec-1), which targets RIP1 (21–24) resulting in caspase-independent cell death. Induction of necroptosis by SHK was reported in human hematopoietic cells (25,26). However, no study has examined the potential for SHK-mediated induction of necroptosis in MM cells.

In this study, we investigated the mechanisms underlying SHK regulation of cell death in MM cells, including apoptosis and necroptosis.

## Materials and methods

### MM cell lines and patient samples

Human myeloma cell lines KMS-12-PE (27), KMS-12-BM (27), RPMI-8226 (28), KMM1 (29), U266 (30), KMS11 (31), and the bortezomib-resistant myeloma cell line KMS11/BTZ (32), were cultured in RPMI-1640 medium containing 10% fetal bovine serum at 37 °C under 5% CO_2_. KMS11/BTZ cells were kindly provided by Kyowa Hakko Kirin Co. Ltd. Bone marrow sample was obtained from an MM patient treated at Kumamoto University Hospital clinically refractory to both bortezomib and lenalidomide under written informed consent. After isolation of mononuclear cells from bone marrow samples using Ficoll-Paque Plus (GE Healthcare, Uppsala, Sweden), myeloma cells were purified using CD138-immunomagnetic beads (Miltenyi Biotech, Paris, France) as previously described (33).

### Reagents

Shikonin, Necrostatin-1 and thapsigargin were purchased from Sigma-Aldrich (St. Louis, MO, USA). VER-155008, an ATP-derivative inhibitor of HSP70, was purchased from Enzo Life Sciences (Farmingdale, NY, USA). These reagents were dissolved in phosphate-buffered saline. Bortezomib was purchased from Janssen Pharmaceutical (Tokyo, Japan).

### Cell viability assay

Cell viability was determined by WST-8 assay using the Cell Counting Kit-8 (Dojindo, Kumamoto, Japan). Briefly, cells were seeded in 96-well plates at a concentration of 2×10^4^/100 μl and incubated with reagents SHK, bortezomib or VER-155008, for 24 h. Following treatment, cells were incubated with WST-8 reagent for a subsequent 3 h. The light absorbance of each well was measured at 450 nm using a VMax absorbance microplate reader (Molecular Devices, Sunnyvale, CA, USA). Data were obtained from three independent experiments.

### Analysis of apoptosis and necroptosis

Cells were incubated at a concentration of 5×10^5^/ml in the presence of SHK for 7 h, or with bortezomib or VER-155008 for 24 h. Cell death was evaluated using the trypan blue exclusion assay (Gibco, Carlsbad, CA, USA). Inhibitors of pan-caspase, Z-VAD-FMK (MBL, Nagoya, Japan) at a concentration of 50 μM, and necroptosis, Nec-1 (necrostatin-1) at a concentration of 60 μM, were employed to distinguish apoptosis and necroptosis, respectively. In some experiments, MM cells were pretreated for 20 min with Z-VAD-FMK before analysis of cell death. Morphological examinations of cells were performed with May-Giemsa staining and transmission electron microscopy. For transmission electron microscopy, cells were centrifuged at 2,000 g for 10 min, fixed in 2.5% glutaraldehyde buffer (pH 7.4, 4°C), and then post-fixed in 1% osmium tetraoxide and embedded in Epon. Ultrathin sections were cut with an ultramicrotome, stained with uranyl acetate and lead citrate, and examined in a Hitachi H-7500 (Tokyo, Japan).

### Western blot analysis

Antibodies against caspase-3, HSP70, HSP90, ubiquitinated proteins and actin were purchased from Santa Cruz Biotechnology (Santa Cruz, CA, USA). Antibodies against caspase-8 and RIP-1 were purchased from Cell Signaling Technology (Beverly, MA, USA). Anti-HSP70 (HSP72) antibody was purchased from Enzo Life Sciences. Cell lysates were prepared using the M-PER mammalian protein extraction reagent (Thermo Scientific Inc., Rockford, IL, USA) after addition of Halt EDTA-free phosphatase inhibitor cocktail and Halt protease inhibitor cocktail (both from Thermo Scientific Inc.). The cell lysates were separated in NuPAGE Bis-Tris precast gels (Invitrogen, Carlsbad, CA, USA) and transferred to PVDF membranes using an iBlot Dry Blotting system (Invitrogen). The membranes were blocked with 5% non-fat dry milk dissolved in Tris-buffered saline (TBS) containing 0.5% Tween-20 (TBS-T) for 1 h at room temperature, followed by incubation with the primary antibodies at 4°C overnight. After washing with TBS-T, the membranes were incubated with a horseradish peroxidase-conjugated secondary antibody (Amersham Biosciences, Oxford, UK) diluted in TBS-T for 2 h at room temperature. The antibody-bound proteins were visualized using an ECL plus kit (Amersham Bioscience).

### Proteasome inhibition assay

Proteasome activity was analyzed by 20S proteasome activity assay kit (Chemicon USA & Canada, cat. no. APT280) according to the manufacturer’s protocol using Corona multi-microplate reader MTP-800AFC (Ibaragi, Japan). Data were obtained from three independent experiments.

### Reverse transcription-polymerase chain reaction (RT-PCR)

RNA was extracted using TRIzol reagent (Invitrogen). cDNA synthesis was performed using the SuperScript III First-Strand Synthesis system for RT-PCR (Invitrogen) according to the manufacturer’s protocol. Thapsigargin, a sarcoplasmic/endoplasmic reticulum Ca^2+^ ATPase (SERCA) inhibitor, was used as an ER stress inducer. ER stress was assessed by detecting activated *XBP-1*, which was analyzed by digestion of PCR products with ApaLI (New England Biolabs Inc., MA, USA), as previously described (34). Because the ApaLI site in *XBP-1* mRNA is spliced out upon activation, activated *XBP-1* shows one large band after ApaLI digestion, while inactivated *XBP-1* shows two ApaLI-digested bands.

Primers for *XBP-1* were 5′-AAA CAG AGT AGC AGC TCA GAC TGC-3′ (sense) and 5′-CTC CCA GAG GTC TAC CCA GAA GGA -3′ (antisense). *GAPDH* was used as a normalization control. Primers for *GAPDH* were previously described (35).

### Statistical analysis and drug combination analyses

Statistical analyses were examined using Student’s t-test. P-values <0.05 were considered statistically significant. The interactions between SHK and VER-155008 was analyzed by Chou’s combination index (CI) using CalcuSyn software Version 2.1 (Biosoft, Cambridge, UK) to determine whether the combination was additive or synergistic (36).

## Results

### SHK induces cytotoxicity in MM cells

**Figure 1:**
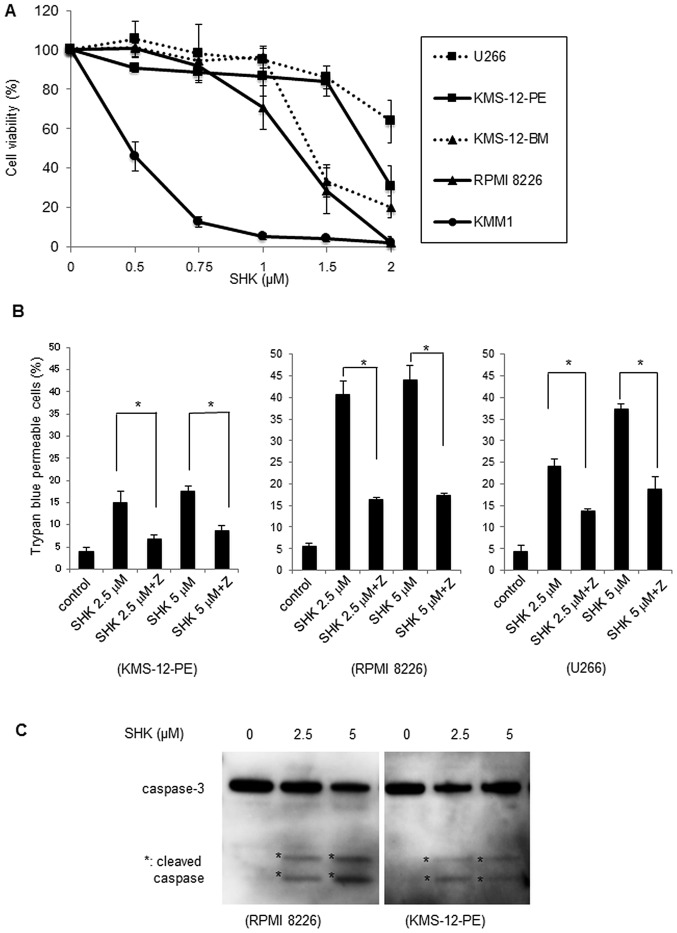

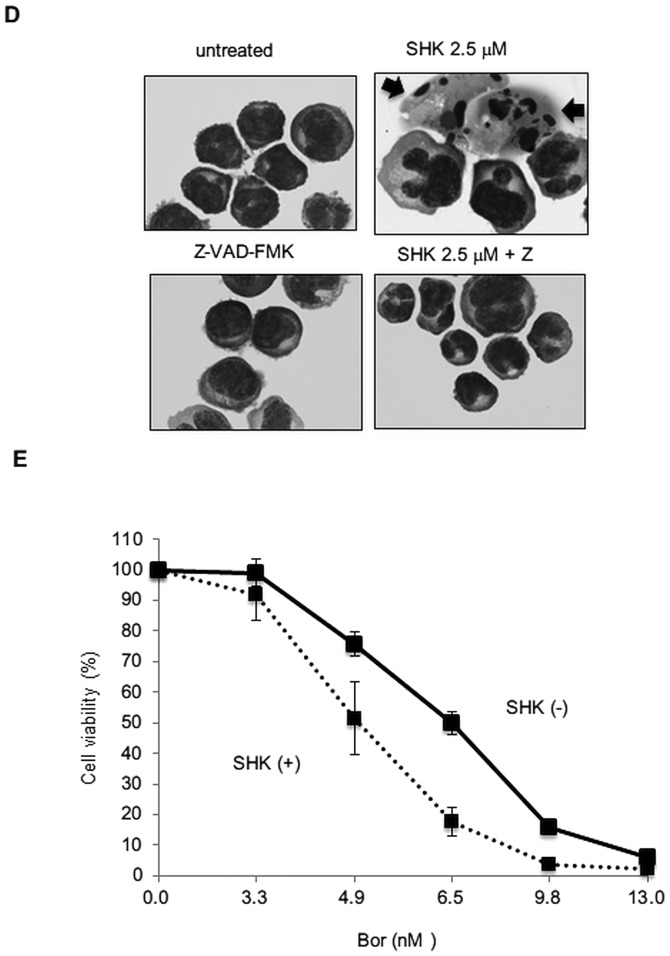
Induction of apoptosis in MM cells by low concentrations of SHK. (A) Five human MM cell lines (U266, KMS-12-PE, KMS-12-BM, RPMI-8226 and KMM1) were cultured for 24 h in the presence of various concentrations of SHK and analyzed by WST-8 assay. All MM cells tested showed dose-dependent cytotoxic effects of SHK. (B) Three representative MM cell lines (KMS-12-PE, RPMI-8226 and U266) were incubated with SHK at 2.5 or 5 μM for 7 h with or without 20 min pre-treatment with Z-VAD-FMK (indicated as Z) and then analyzed by trypan blue dye exclusion assay. SHK-induced cell death was partly inhibited by Z-VAD-FMK. ^*^P<0.01. (C) Western blot analyses of caspase-3. SHK activated caspase-3 at concentrations of 2.5 and 5 μM for 7 h. (D) Morphological changes of MM cells after incubation with SHK. KMS-12-PE cells were treated with 2.5 μM SHK for 5 h either with or without pre-treatment of Z-VAD-FMK and evaluated by cytospin analysis. Cells were stained with May-Giemsa staining solution. SHK clearly induced apoptotic morphological changes, such as fragmented nucleus (arrows), and apoptosis was inhibited by Z-VAD-FMK. (E) Enhanced cytotoxic effects of bortezomib by SHK. KMS-12-PE cells were incubated with various concentrations of bortezomib either with (dotted line) or without 0.5 μM SHK (solid line). Cell viability was analyzed by WST-8 assay. Marked sensitization of MM cells to bortezomib by SHK was observed.

The human MM cell lines were cultured for 24 h in the presence of various concentrations of SHK and cell viability was analyzed by WST-8 assay. As shown in Fig. 1A, SHK exerted cytotoxic effects in all MM cells in a dose-dependent manner, although the effect was varied. These results clearly indicated that SHK exhibits cytotoxicity in MM cells at concentrations <2 μM.

### SHK induces apoptosis in MM cells

To further investigate the mechanisms of SHK in regulating cell death, we utilized the pan-caspase inhibitor Z-VAD-FMK. After pretreatment of three MM cell lines, KMS-12-PE, RPMI-8226 and U266, with Z-VAD-FMK for 20 min, cells were incubated with SHK at 2.5 or 5 μM for 7 h and then analyzed by trypan blue assay. As shown in Fig. 1B, the percentages of trypan blue permeable cells under treatment with SHK were partly inhibited by treatment with Z-VAD-FMK (P<0.01), indicating that SHK induced cell death in MM cells via activated caspases.

Western blot analysis revealed that caspase-3 was activated by SHK (Fig. 1C). Morphological analysis of SHK-treated MM cells showed typical apoptotic changes, such as decreased cellular volume, chromatin condensation, and nuclear fragmentation, and these apoptotic morphological changes were inhibited by Z-VAD-FMK treatment (Fig. 1D). Together these observations suggest that SHK induces apoptosis in MM cells.

In combination with bortezomib, SHK increased cytotoxic effects for KMS-12-PE cells at a concentration of 0.5 μM (Fig. 1E), a concentration at which SHK alone was unable to induce cytotoxic effects (Fig. 1A). This indicates that low dose SHK sensitizes MM cells to bortezomib.

### SHK induces apoptosis in bortezomib-resistant cells and freshly isolated MM cells

**Figure 2:**
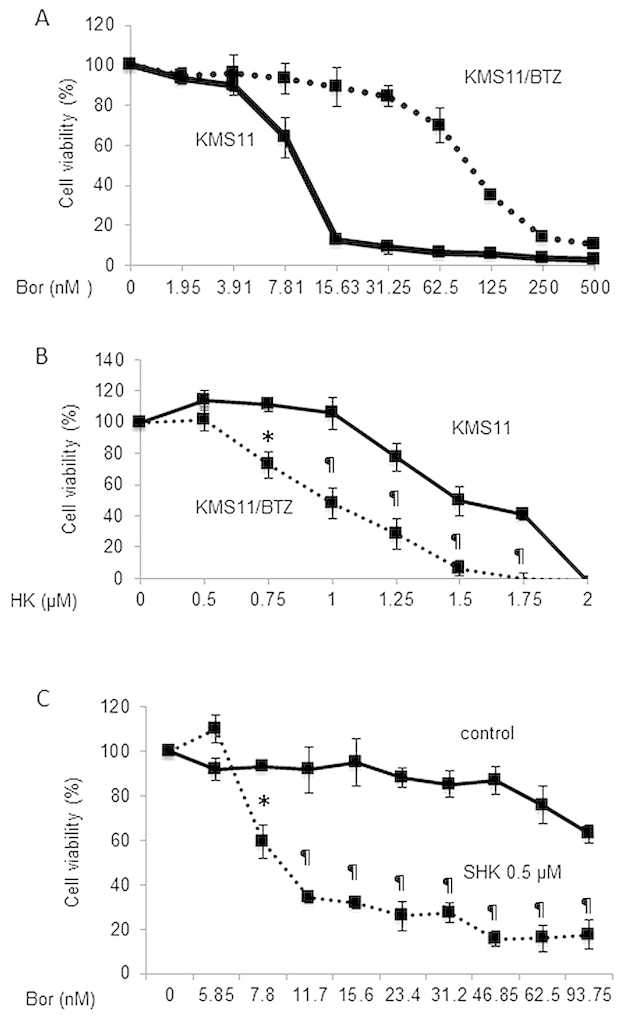

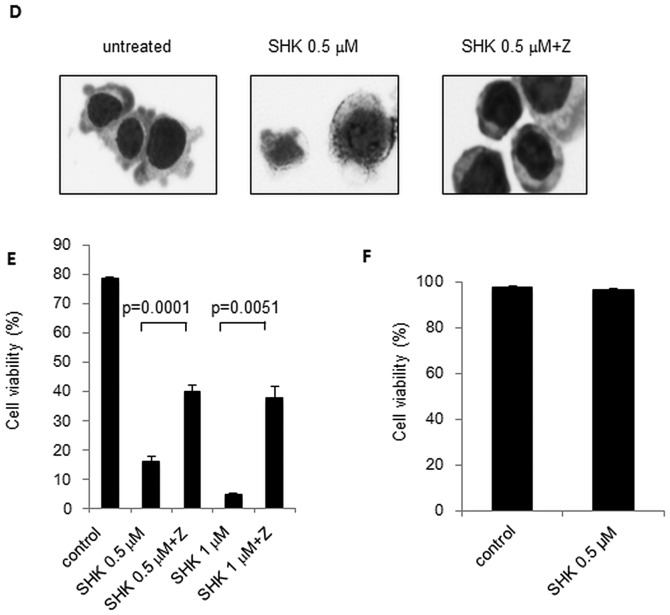
Antitumor effect of SHK on bortezomib resistant cells, primary MM cells and peripheral blood mononuclear cells. The bortezomib resistant MM cell line KMS-11/BTZ, and the parental cell line KMS11, were cultured with various concentrations of bortezomib (A), SHK (B) or in combination (C) for 24 h and cell viabilities were analyzed by WST-8 assay. (A) The bortezomib resistant MM cell line KMS-11/BTZ (dotted line), and the parental cell line, KMS11 (solid line), were treated with bortezomib for 24 h and subsequently analyzed by WST8 assay. The IC_50_ values of the KMS11 and KMS11/BTZ cells were 9.9 and 98.5 nM, respectively. (B) The IC_50_ value of SHK for KMS11/BTZ cells (dotted line) was even lower than that of KMS11 cells (solid line) (1.1 vs. 1.56 μM, respectively). ^*^P<0.05, ^¶^P<0.01. (C) Treatment of KMS11/BTZ with (dotted line) or without (solid line) low concentration of SHK (0.5 μM), which alone does not show cytotoxic effects, increased the sensitivity to bortezomib. ^*^P<0.005, ^¶^P<0.0001. (D and E) MM cells from primary bone marrow sample were incubated with 0.5 μM SHK for 16 h and then either evaluated by cytospin analysis (D) or WST-8 assay (E). Both analyses revealed marked increase of dead cells in response to treatment with SHK and inhibition by Z-VAD-FMK. (F) Lack of cytotoxic effect of SHK in normal PBMCs. PBMCs were cultured with 0.5 μM SHK for 24 h and evaluated by trypan blue dye exclusion assay. There was no increase of dead cells by SHK.

We further investigated whether SHK can overcome resistance to bortezomib. We used the bortezomib resistant MM cell line KMS-11/BTZ, which has a 9.9-fold higher IC_50_ value to bortezomib than that of its parental cell line KMS11 (Fig. 2A). As shown in Fig. 2B, viability of both KMS11 and KMS-11/BTZ cells was inhibited by SHK in a dose-dependent manner. Interestingly, the IC_50_ value of SHK for KMS11/BTZ cells was even lower than that for KMS11 cells (1.1 vs. 1.56 μM, respectively). These results suggest that SHK may overcome refractoriness of MM cells to bortezomib. We then examined the effects of a combination of SHK and bortezomib on KMS11/BTZ cells. As shown in Fig. 2C, treatment of KMS11/BTZ cells with very low concentration of SHK (0.5 μM) significantly increased the sensitivity to bortezomib, suggesting re-sensitization of bortezomib-resistant MM cells to bortezomib by SHK.

We further investigated whether SHK could induce cell death in primary MM cells from a patient clinically refractory to both bortezomib and lenalidomide. We observed marked cell death in response to treatment with SHK, and the proportion of dead cells was clearly inhibited by Z-VAD-FMK (Fig. 2D and E) while SHK alone did not influence viability of peripheral blood mononuclear cells (PBMCs) from a healthy donor (Fig. 2F). Together this suggests that SHK exerts anti-tumor effects in primary MM cells refractory to bortezomib while sparing toxicity to normal PBMCs.

### SHK inhibits proteasome functions and induces ER stress response

**Figure 3:**
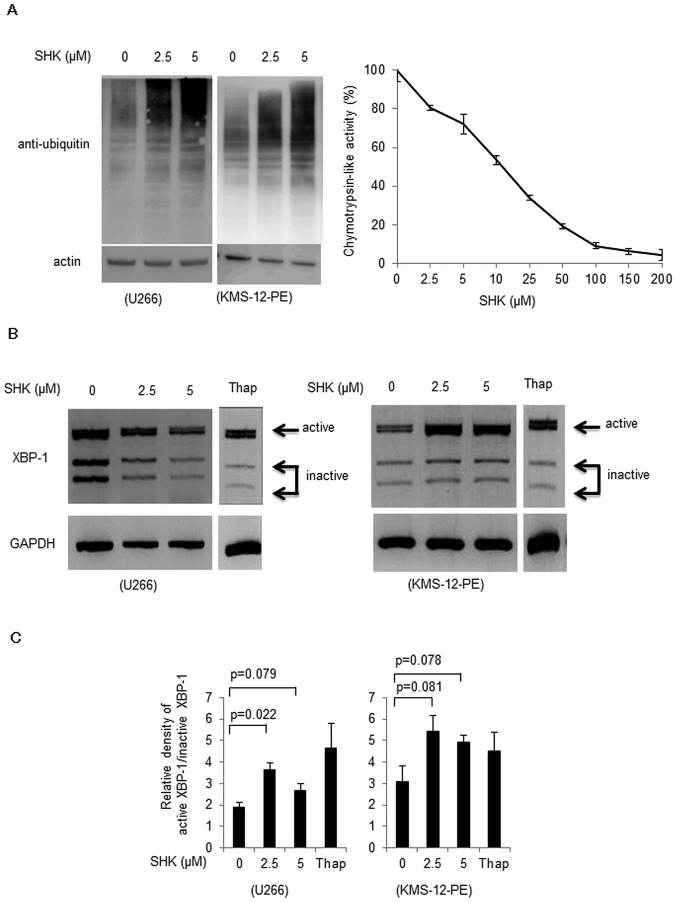
Accumulation of ubiquitinated proteins and activation of XBP-1 by SHK. (A) Left panel, western blot analyses of ubiquitinated proteins. U266 and KMS-12-PE cells were incubated with 2.5 or 5 μM SHK for 7 h. SHK induced an accumulation of ubiquitinated proteins in a dose-dependent manner. Right panel, inhibition of 20S chymotrypsin-like activity by SHK. SHK decreased 20S chymotrypsin-like activity at a dose-dependent manner. (B) Activation of *XBP-1* by SHK. XBP-1 mRNA from U266 and KMS-12-PE cells treated with SHK was converted to cDNA by RT-PCR and then subsequently digested with ApaLI to distinguish inactive and active *XBP-1*. Thapsigargin (Thap) was used as an endoplasmic reticulum stress inducer at 100 nM for 8 h. SHK was used at concentrations of 2.5–5 μM for 2 h. The longer fragment derived from the active form of XBP-1 mRNA (upper band) and two shorter bands derived from the inactive form (middle and lower bands) were detected. A decrease of the inactive bands was found in U266 (left panel) cells, while increase of active band was noted in KMS-12-PE cells (right panel) by treatment with SHK. (C) Relative density of active XBP-1 compared with inactive XBP-1, as shown in (B) was calculated. A significant increase of active XBP-1 was found by treatment with SHK in U266 cells. KMS-12-PE cells showed a similar trend, although the difference was not statistically significant.

We further analyzed in detail the mechanisms underlying SHK-induced apoptosis. A previous report (11) suggested possible proteasome inhibitor function of SHK, and we also found that SHK induced an accumulation of ubiquitinated proteins in a dose-dependent manner (Fig. 3A, left panel). To further confirm proteasome inhibitory mechanisms by SHK, direct interaction between SHK and 20S proteasome was analyzed. We found that SHK inhibited proteasome enzymatic activity at a dose-dependent manner (Fig. 3A, right panel).

As shown in Fig. 3B and C, SHK induced spliced form of XBP-1 in both U266 cells and KMS-12-PE cells. These results suggest that SHK may function as a proteasome inhibitor, which eventually evokes the ER stress response.

**Figure 4:**
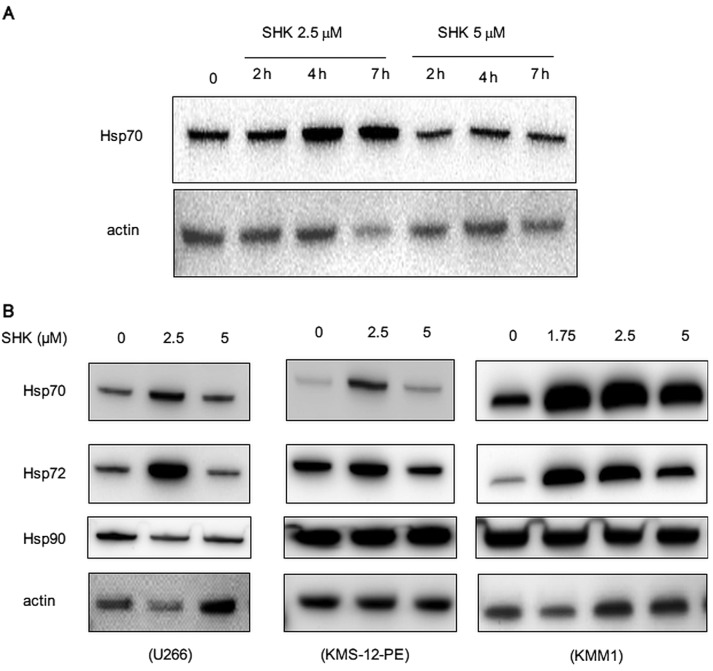

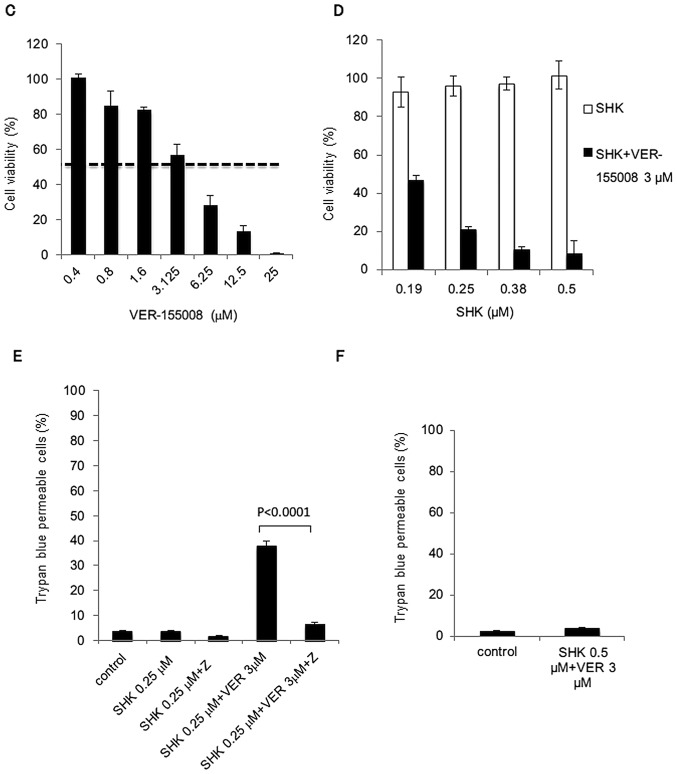
Increase of HSP70/72 by SHK and synergistic cytotoxic effects of SHK in combination with HSP70/72 inhibitor. (A) Western blot analyses of HSP70. SHK at a concentration of 2.5 μM transiently increased HSP70 in KMS-12-PE cells in a time-dependent manner, while this was less evident at 5 μM. (B) Western blot analyses of HSP70, HSP72, and HSP90. U266, KMS-12-PE and KMM1 were treated with 2.5 and 5 μM SHK for 7 h. Induction of HSP70 and HSP72 by SHK was observed in all cell lines and maximized at 2.5 μM. There was no change in the expression of HSP90. (C) Cytotoxic effect of the HSP70/72 inhibitor VER-155008 in KMS-12-PE cells. Cells were cultured with various concentrations of VER-155008 for 24 h and evaluated by WST-8 analysis. VER-155008 alone induced cytotoxic effects in MM cells. Note that VER-155008 at ~3 μM showed 55% growth inhibition (dotted line). (D) Combination effects of VER-155008 and SHK. KMS-12-PE cells were treated with SHK at concentrations varying from 0.19 to 0.5 μM either with 3 μM VER-155008 (solid bars) or SHK alone (blank bars) for 24 h. Combinations of SHK and VER-155008 showed significant synergistic effects in induction of cytotoxicity (CI=0.72). (E) The populations of dead cells induced by the combination of SHK and VER-155008 (VER) were partly inhibited by Z-VAD-FMK (P<0.0001). (F) Combination of SHK and VER-155008 did not show toxic effects in normal PBMCs. PBMCs from a normal donor were cultured with SHK and VER-155008 (VER) at 0.5 and 3 μM, respectively, for 24 h and evaluated by trypan blue dye exclusion analysis. No cytotoxic effect was observed.

SHK increases HSP 70/72 and exerts cytotoxic effect in combination with HSP70/72-inhibitor, VER-155008. Our preliminary observations using mass-spectrometry analysis suggested induction of heat shock proteins (HSPs) by SHK (data not shown), which was consistent with previous reports in monocytes and leukemia cells (13,14). We next analyzed if SHK induced HSPs in MM cells. As expected, SHK transiently increased amounts of HSP70 after 2–7 h of treatment (Fig. 4A). This was more prominent at a concentration of 2.5 μM than 5 μM, suggesting that induction of HSP70 is dose-sensitive. We also detected a similar induction of HSP72, an inducible form of HSP70, while there was no significant change in expression of HSP90 (Fig. 4B).

Because HSPs are known to support cell survival under various stresses, we utilized the HSP70/72 inhibitor VER-155008, to analyze the influence of HSPs in cytotoxic effects delivered by SHK. VER-155008 alone increased cytotoxicity of MM cells in a dose-dependent manner (Fig. 4C), indicating that inhibition of constitutive HSPs is capable of induction of cell death. Interestingly, addition of VER-155008 to cells treated with low dose SHK prepared at concentrations <0.5 μM enhanced the proportion of dead cells, whereas SHK alone did not influence cell viability (Fig. 4D). The combination index analysis (CI=0.72) according to the method of Chou (36) revealed a synergistic effect of SHK and VER-155008. The proportion of dead cells induced by the combination of SHK and VER-155008 was partly reduced by treatment with Z-VAD-FMK, indicating that the combination of SHK and VER-155008 induced caspase-mediated apoptosis (Fig. 4E). Analysis using PBMCs from a normal donor showed no cytotoxic effect by this combination treatment (Fig. 4F).

### SHK induces necroptosis in MM cells at high concentrations

**Figure 5:**
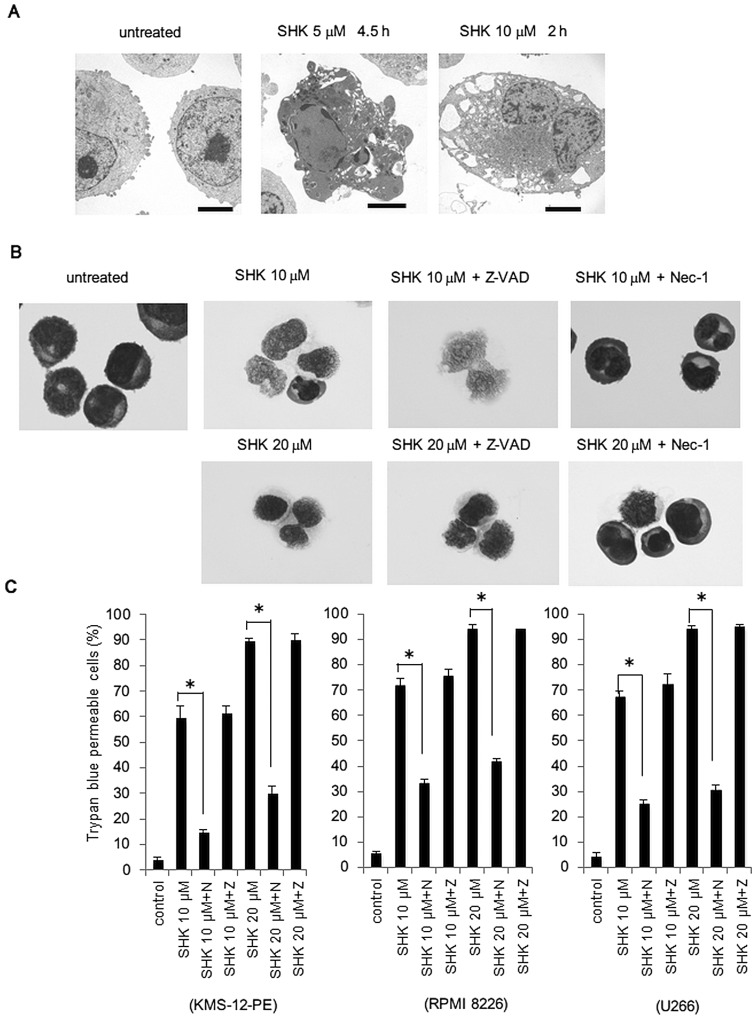

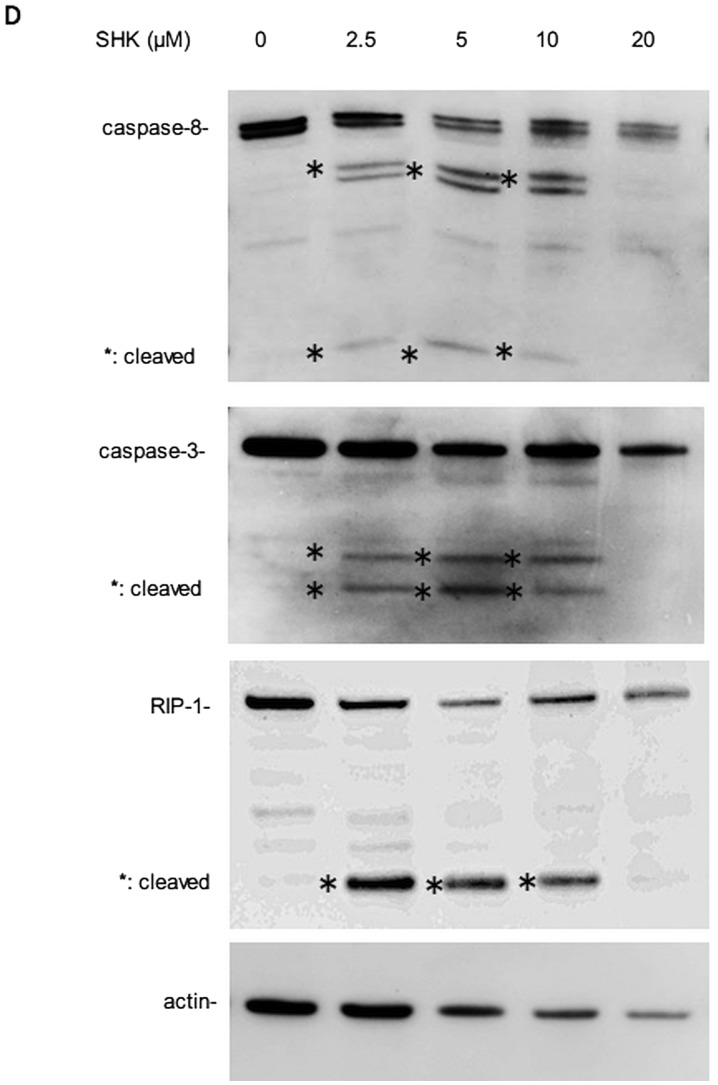
Induction of necroptosis in MM cells by SHK. (A) Electron microscopic examination of KMS-12-PE cells treated with 5 μM SHK for 4 h showed typical apoptotic changes, such as fragmented and condensed nuclei (middle panel). In contrast, treatment of 10 μM SHK for 2 h induced typical necrotic changes, such as translucent cytoplasm and swelling of cell membranes (right panel). Scale bar, 5 μm. (B) Morphological changes of KMS-12-PE cells after incubation with 10 or 20 μM SHK for 5 h. SHK induced ghost cells (left panel) and this was inhibited by treatment with Nec-1 (right panel). No apparent inhibition of cell death was found by Z-VAD-FMK treatment (middle panel). (C) Nec-1 inhibited cell death induced by SHK. KMS-12-PE, RPMI-8226 and U266 cells were incubated with 10 or 20 μM SHK in the presence or absence of Nec-1 (indicated as N) or Z-VAD-FMK (indicated as Z) for 7 h and then analyzed by trypan blue dye exclusion assay. SHK-induced cell death was significantly inhibited by Nec-1 (P<0.01) and not affected by Z-VAD-FMK (^*^P<0.01). (D) Western blot analyses of caspase-8, -3, and RIP-1. RPMI-8226 cells were treated with SHK at concentrations from 2.5 to 20 μM. SHK activated caspase-8 and -3 at concentrations <10 μM while no changes were detected at 20 μM SHK. RIP-1 was cleaved by SHK <10 μM and remained intact at 20 μM.

Because SHK has been reported to induce necroptosis in various tumor cell lines, we examined whether SHK could induce necroptosis in MM cells. As shown in Fig. 5A, electron microscopic examination revealed typical necrotic changes, such as translucent cytoplasm and swelling of cell membranes by treatment with SHK at a concentration of 10 μM for 2 h, while apoptotic changes were observed with treatment with SHK at lower concentrations. To further confirm the induction of necroptosis, cells were either treated with Z-VAD-FMK or the necroptosis inhibitor, necrostatin-1 (Nec-1), in combination with SHK at 10 and 20 μM. As shown in Fig. 5B, cell death was completely inhibited by (Nec-1), and unaffected by Z-VAD-FMK, indicating that cell death delivered by SHK at high concentration was necroptosis. Trypan blue dye exclusion assay showed that SHK greatly increased the proportion of dead cells at 10 or 20 μM, and this was again inhibited by Nec-1 and not affected by Z-VAD-FMK (Fig. 5C), confirming the induction of necroptosis by high-dose SHK. Western blot analysis revealed activation of caspase-8 and -3 by SHK at concentrations starting from 2.5 μM and maximizing at 5 μM, and decreasing at higher concentrations (Fig. 5D, upper panels), indicating that activation of caspases are specifically dependent on concentration of SHK. Because necroptosis is dependent on RIP1 function, we further evaluated the status of RIP1 in response to SHK. We detected cleavage of RIP1 by treatment with SHK at lower concentrations (~10 μM), while it was not cleaved and remained active at higher concentrations (Fig. 5D, lower panel), indicating that SHK dynamically regulates the cleavage of RIP1 in a concentration-dependent manner.

## Discussion

Our above results demonstrate that SHK exerted antitumor effects in MM cells. No previously examination of the potential cytotoxic effects of SHK in MM cells, has been reported and to the best of our knowledge, ours is the first report providing possible therapeutic efficacy of SHK in MM. We also showed that SHK exerted cytotoxicity to both the bortezomib-resistant cell line and freshly isolated MM cells from a patient clinically refractory in both bortezomib and lenalidomide. The IC_50_ value of SHK to a bortezomib-resistant cell line was even lower than that of the parental cell line. The mechanisms regulating cell death in bortezomib resistant cell line is unknown. Western blot analysis of KMS11/BTZ treated with SHK revealed slight accumulation of ubiquitinated proteins but the amount was rather less than what found in parental cells (data not shown), suggesting SHK should possess other mechanisms than proteasome inhibition. Collectively, these findings suggested that monotherapy using SHK may overcome refractoriness to bortezomib. Alternatively, since treatment of the bortezomib resistant MM cell line KMS-11/BTZ, with very low concentration of SHK (0.5 μM) sensitized cells to bortezomib; this suggests that SHK may be utilized as a combinational reagent with bortezomib. These results indicate that SHK may overcome refractoriness of MM cells to bortezomib by either monotherapy or in combination with bortezomib. Future studies in other refractory cases should be performed to examine this possibility.

Next, we attempted to elucidate the biological mechanisms of SHK in inducing cell death. Because previous reports showed induction of HSP70 (13,14) or inhibition of proteasome activity by SHK (11), and our preliminary observations using mass-spectrometry analysis suggested induction of HSPs by SHK (data not shown), we investigated whether SHK had an effect on HSP70 or ubiquitinated proteins. Accumulation of ubiquitinated proteins and activation of XBP-1 were found in response to treatment with SHK at low concentration (<5 μM), suggesting that SHK may function as a proteasome inhibitor eventually leading to accumulation of ER stress. This ER stress inducing property of SHK may explain the coordinate action of SHK and bortezomib in respect to augmented inhibition of proteasome function.

That HSP70 induction is detected by SHK treatment may seem conflicting, as SHK alone causes cytotoxic effects and HSP70 is considered to support cell survival. HSP70 is overexpressed in MM cells and HSP70 inhibition is reported to reverse drug resistance (15–17). Our observation that transient increase of both HSP70 and its inducible form of HSP70 (HSP72) by SHK may be explained by accumulation of ER stress by SHK, although the exact mechanisms underlying SHK regulation of HSP70 is not known. Although SHK eventually induces apoptosis, cells may try to overcome these stress by inducing molecules such as heat shock proteins. In that situation, imbalance of stress and stress-relief should be a key for survival of tumor cells.

To evaluate if antitumor effects delivered by SHK could be enhanced by HSP70 inhibition, a combination of SHK and the HSP70 inhibitor VER-155008 was used. As expected, VER-155008 alone exerted cytotoxicity to MM cells in a dose-dependent manner. This was not surprising because HSP inhibitors are reported to be therapeutic candidates for MM (37,38). However, combination of VER-155008 at 3 μM, which exerts ~40% reduction of cell viability, with SHK at very low concentration (<0.5 μM) significantly increased the proportion of dead cells. These results show, for the first time, that besides SHK alone potentially serving as a treatment approach in MM, it significantly shows efficacy in inhibiting growth of MM cells in combination with the HSP70 inhibitor. Although efficacy of SHK may be limited, combining HSP70 inhibitor with SHK may promise efficient induction of cell death. We found that the concentrations of SHK required for combined treatment with HSP70 inhibitor is quite low, allowing us to minimize the toxic effect of SHK to normal tissues. Indeed, both SHK alone at low concentrations and in combination with VER-155008 did not show toxicity to normal PBMCs, suggesting the safety of the combination. However, extensive analysis regarding toxicity of SHK to normal tissues should be performed before clinical utilization.

Furthermore, we showed that SHK at higher concentrations induced necroptosis in MM cells. Necroptosis (programmed necrosis) is regulated by RIP1 and RIP3 through complex formation and activation (20,21,39). Necroptosis, which is distinct from apoptosis, exhibits specific characteristics. First, after the death receptor family is stimulated, necroptosis is induced under activation of RIP by inhibition of caspase-8, resulting in caspase-independent cell death (24). By contrast, under activation of caspase-8, it cleaves RIP1 and RIP3, resulting in inhibition of necroptosis and induction of apoptosis. Second, Nec-1, an inhibitor of RIP-kinase-1, specifically inhibits necroptosis (22). Third, translucent cytoplasm and swelling of cell membranes are morphological characteristics of necroptosis, and distinct from those in apoptosis, such as condensed chromatin and fragmented nucleus. In the present report, because activation of caspase-8 and -3 followed by cleavage of RIP1 were found by treatment with SHK at lower concentrations, this suggests that mechanisms required for induction of necroptosis is disrupted by SHK at its low concentration. On the other hand, SHK did not activate caspase-3 and caspase-8 at high concentrations, allowing RIP-1 to remain in an intact form, which leads to induction of necroptosis. Thus, our findings indicate apoptosis and necroptosis are strictly regulated, depending on the amount of SHK.

Taken together, our results show that SHK at low concentrations may have a potential role as an inducer of apoptosis in MM cells, including drug resistant clones, through affecting caspases. SHK shows potential to be a new therapeutic agent for treating MM in combination with HSP70 inhibitors. Moreover, SHK at high concentrations may have a potential role as an inducer of necroptosis in MM cells. Since necroptosis has not been considered as a therapeutic strategy in treating MM, this approach might serve as a new modality leading to better control of MM cells. Elucidating the mechanisms underlying the multiple effects of SHK such as reversal of bortezomib resistance and induction of necroptosis by determination of molecules targeted by SHK is currently underway.
